# Linking hierarchical classification of transcription factors by the structure of their DNA-binding domains to the variability of their binding site motifs

**DOI:** 10.18699/vjgb-25-99

**Published:** 2025-12

**Authors:** V.G. Levitsky, T.Yu. Vatolina, V.V. Raditsa

**Affiliations:** Institute of Cytology and Genetics of the Siberian Branch of the Russian Academy of Sciences, Novosibirsk, Russia Institute of Molecular and Cellular Biology of the Siberian Branch of the Russian Academy of Sciences, Novosibirsk, Russia; Institute of Molecular and Cellular Biology of the Siberian Branch of the Russian Academy of Sciences, Novosibirsk, Russia; Institute of Cytology and Genetics of the Siberian Branch of the Russian Academy of Sciences, Novosibirsk, Russia

**Keywords:** de novo motif search, motifs of transcription factor binding sites, structural variants of motifs of transcription factor binding sites, cooperative action of transcription factors, similarity of motifs of transcription factor binding sites, massive whole-genome sequencing of transcription factor binding sites, de novo поиск мотивов, мотивы сайтов связывания транскрипционных факторов, структурные варианты мотивов сайтов связывания транскрипционных факторов, сходство мотивов сайтов связывания транскрипционных факторов, кооперативное действие транскрипционных факторов, массовое полногеномное секвенирование сайтов связывания транскрипционных факторов

## Abstract

De novo motif search is the main approach for determining the nucleotide specificity of binding of the key regulators of gene transcription, transcription factors (TFs), based on data from massive genome-wide sequencing of their binding site regions in vivo, such as ChIP-seq. The number of motifs of known TF binding sites (TFBSs) has increased several times in recent years. Due to the similarity in the structure of the DNA-binding domains of TFs, many structurally cognate TFs have similar and sometimes almost indistinguishable binding site motifs. The classification of TFs by the structure of the DNA-binding domains from the TFClass database defines the top levels of the hierarchy (superclasses and classes of TFs) by the structure of these domains, and the next levels (families and subfamilies of TFs) by the alignments of amino acid sequences of domains. However, this classification does not take into account the similarity of TFBS motifs, whereas identification of valid TFs from massive sequencing data of TFBSs, such as ChIP- seq, requires working with TFBS motifs rather than TFs themselves. Therefore, in this study we extracted from the Hocomoco and Jaspar databases the TFBS motifs for human and fruit fly Drosophila melanogaster, and considered the pairwise similarity of binding site motifs of cognate TFs according to their classification from the TFClass database. We have shown that the common tree of the TF hierarchy by the structure of DNA-binding domains can be split into separate branches representing non-overlapping sets of TFs. Within each branch, the majority of TF pairs have significantly similar binding site motifs. Each branch can include one or more sister elementary units of the hierarchy and all its/their lower levels: one or more TFs of the same subfamily, or the whole subfamily, one or several subfamilies of the same family, an entire family, etc., up to the entire class. Analysis of the seven largest human and two largest Drosophila TF classes showed that the similarity of TFs in terms of TFBS motifs for different corresponding levels (classes, families) is noticeably different. Supplementing the hierarchical classification of TFs with branches combining significantly similar motifs of TFBSs can increase the efficiency of identifying involved TFs through enriched motifs detected by de novo motif search for massive sequencing data of TFBSs from the ChIP-seq technology.

## Abstract (ru)

Поиск мотивов de novo – базовый подход определения нуклеотидной специфичности связывания важнейших регуляторов транскрипции генов, транскрипционных факторов (ТФ), на основе данных массового полногеномного секвенирования районов их сайтов связывания in vivo, таких как ChIP-seq. Количество известных мотивов сайтов связывания ТФ (ССТФ) возросло в несколько раз в последние годы. Из-за сходства структуры ДНК-связывающих доменов ТФ многие структурно родственные ТФ имеют сходные или даже неразличимые мотивы сайтов связывания. Классификация ТФ по структуре ДНК-связывающих доменов из базы данных TFClass определяет верхние уровни иерархии (суперклассы и классы ТФ) по структуре этих доменов, а следующие уровни (семейства и подсемейства ТФ) по выравниваниям аминокислотных последовательностей доменов. Однако эта классификация не учитывает сходство мотивов ССТФ, а для идентификации действующих ТФ по данным массового секвенирования ССТФ ChIP-seq приходится иметь дело с мотивами ССТФ, а не с самими ТФ. Поэтому в данной работе мы взяли из баз данных Hocomoco/Jaspar мотивы CCТФ человека/плодовой мушки Drosophila melanogaster и рассмотрели сходство мотивов сайтов связывания в парах родственных ТФ согласно их классификации в базе данных TFClass. Показано, что общее дерево иерархии ТФ по структуре ДНК- связывающих доменов можно разделить на отдельные неперекрывающиеся множества ТФ – ветви. В пределах каждой ветви большинство пар ТФ имеет значимо похожие мотивы сайтов связывания. Каждая ветвь включает одну или несколько сестринских элементарных единиц иерархии и все более низкие ее/их уровни: один или несколько ТФ одного подсемейства или целое подсемейство, одно или несколько подсемейств одного семейства, целое семейство и т. д. до целого класса. Анализ семи крупнейших классов ТФ человека и двух плодовой мушки показал, что сходство ТФ по мотивам ССТФ для разных соответствующих уровней (классов, семейств) заметно отличается. Дополнение иерархической классификации ТФ ветвями, объединяющими значимо сходные мотивы ССТФ, может повысить эффективность идентификации ТФ, вовлеченных в регуляцию транскрипции, по результатам de novo поиска обогащенных мотивов для данных массового секвенирования ССТФ с помощью технологии ChIP-seq.

## Introduction

The study of the regulation mechanisms of eukaryotic genes transcription is necessary for understanding molecular genetic processes in the cell. Gene transcription is carried out under the control of special proteins, transcription factors (TFs), which regulate it specifically by the nucleotide context by binding to genomic DNA (Lambert et al., 2018). This specificity is due to nucleotide sequences of binding sites being recognized by individual TFs (TFBSs). The variability of binding sites reflects the ability of each TF to bind to different DNA sequences; therefore, the set of similar binding site sequences interacting with a TF is called the motif of its binding sites (D’haeseleer, 2006). The length of the region of genomic DNA directly interacting with an individual TF, as well as the length of the TFBS motif, usually vary from 6 to 20 base pairs (bp) (Spitz, Furlong, 2012; Zambelli et al., 2013; Vorontsov et al., 2024). One TF may have several distinct motifs of binding sites. The most popular model of the TFBS motif is the positional weight matrix (PWM). To build a model of the PWM motif, it is necessary to calculate the nucleotide frequencies at all positions using this alignment of the TFBSs representing this motif, and calculate the contributions (or weights) to the total estimate of affinity using these frequencies for each of the four nucleotides at each position. The total estimate of affinity for a potential site in a DNA sequence is equal to the sum of the weights corresponding to the nucleotides encountered, for all its positions (Wasserman, Sandelin, 2004).

Experimental ChIP-seq technology is based on chromatin immunoprecipitation (ChIP), i. e. application of antibodies to the target protein under study, for example, a TF. This technology is used to identify interactions of target proteins with genomic DNA in vivo. The essence of this technology is to perform chromatin immunoprecipitation and subsequently to map the genomic loci of the interaction between a target protein and genomic DNA. TFs in vivo, as a rule, act as part of multiprotein complexes formed by protein-protein interactions of several TFs, which allows them to regulate gene transcription together, even without direct connections of each of the TFs with genomic DNA. Therefore, in vivo TFs can bind to DNA in a variety of ways:

• directly, there is a binding site of the target TF in DNA; • with another “partner” TF, binding sites for both target and partner TFs co-occur in DNA, they are found with a spacer or an overlap (Levitsky et al., 2019); • indirectly, there is a binding site for a partner TF in DNA, and that for the target TF is absent (Slattery et al., 2014).

The individual genomic loci mapped in a ChIP-seq experiment are called peaks and range in length from several hundreds to thousands of bp (Johnson et al., 2007, Nakato, Shirahige, 2017; Lloyd, Bao, 2019). Each of the peaks does not necessarily contain the binding site of the target TF, direct binding can be performed by one of the possible partner TFs. Massive application of other in vivo experimental sequencing technologies besides ChIP-seq, e. g. CUT&RUN (Sken, Henikoff, 2017), as well as in vitro technologies (PBM, HT-SELEX) (Stormo, Zhao, 2010; Jolma et al., 2013; Franco-Zorrilla et al., 2014) allowed to accumulate data on the nucleotide specificity of binding sites of hundreds of TFs for the main model eukaryotic species. Several databases (DBs) performed uniform primary processing of massive genomewide TFBS sequencing data, including ChIP-seq data (GTRD, Kolmykov et al., 2021; ReMap, Hammal et al., 2022; Cistrome DB, Taing et al., 2024).

Enrichment analysis of TFBS motifs, in particular the de novo motif search (Zambelli et al., 2013; Liu et al., 2018; Bailey, 2021), was initially used only to confirm the validity of the results of ChIP-seq experiments (sets of DNA sequences or peaks). Then, the de novo motif search became the standard approach for analysis of peak sets, allowing to determine enriched motifs, presumably corresponding to the motifs of the binding sites of the target TF and several partner TFs, cooperatively acting in the regulation of gene transcription (Spitz, Furlong, 2012; Slattery et al., 2014; Morgunova, Taipale, 2017).

To date, for several hundred TFs of the main eukaryotic taxa, such as mammals, insects and higher plants, TFBS motifs of the PWM model (nucleotide frequency matrices) are compiled in a number of DBs, JASPAR (Rauluseviciute et al., 2024), Hocomoco (Vorontsov et al., 2024) and Cis-BP (Weirauch et al., 2014). For example, the Hocomoco DB (version 12, Vorontsov et al., 2024) amounts to 1,443 binding site motifs for 949 human TFs. The analysis pipeline used by the Hocomoco DB for human and mouse TFBS motifs allowed identifying more than one structural type of motif for several hundred annotated TFs.

For a single TF, both the number of different binding site motifs and the structure and variability of each of the motifs are determined by the structure of the DNA binding domain (DBD) of this TF (Wingender, 1997, 2013). Based on the analysis of the similarity of the structure of DBDs of TFs and the alignment of the amino acid sequences of DBDs of TFs, a hierarchical classification of TFClass was developed, first for human TFs, and then for their orthologs in rodents and mammals (Wingender et al., 2013, 2015, 2018). This classification has six hierarchy levels. The upper levels of the hierarchy, superclass and class are defined according to the general topology and structural features of the DBDs of TFs. The next levels of the family and subfamily are deduced by the similarity of amino acid sequences of DBDs of TFs based on their alignments. The lower levels are the TF gene and the structural variant of its protein. In total, mammals have nine superclasses. Analysis of the structure of DBDs of TFs in plants did not reveal additional superclasses, however, about half of the TF classes turned out to be plant-specific (Plant- TFClass DB, Blanc-Mathieu et al., 2024).The most important function of TFs in vivo is their ability to bind DNA specifically. However, the TFClass classification does not take into account the similarity of TFBS motifs at certain hierarchy levels, in specific classes, families, etc. The similarity of TFBS motifs can vary greatly in different classes of TFs. For example, the largest class of mammalian TFs, C2H2 zinc finger factors {2.3}, has the most noticeable variability in TFBS motifs (Najafabadi et al., 2015; Lambert et al., 2018). Hereinafter, numbers in curly brackets denote the TF classification nomenclature from the TFClass (Wingender, 1997, 2013; Wingender et al., 2013, 2015, 2018). For example, TF JUN belongs to the superclass Basic domains {1}, the class Basic leucine zipper factors (bZIP) {1.1}, the Jun-related family {1.1.1}, and the Jun subfamily {1.1.1.1}. To determine a functioning TF by a given enriched motif of its binding sites as a result of a de novo motif search, we can apply not only the classification of TFs by the structure of their DBDs but also the classification of TFs by the similarity of TFBS motifs.

An important step in the analysis of the results of de novo enriched motif search applied for ChIP-seq data is the most precise determination of the motifs of binding sites of target and partner TFs based on the enriched motifs obtained. A common way to limit the list of putative TFs for each enriched motif is to assess the significance of its similarity to the TFBS motifs of known TFs from the DBs (Weirauch et al., 2014; Rauluseviciute et al., 2024; Vorontsov et al., 2024). Standard tools such as TomTom (Gupta et al., 2007) can be used to assess similarity in the pairs of motifs of the PWM model.

The estimate of the total number of human TFs is 1,659 (Shen et al., 2023); however, both the number of structurally different DBDs of TFs and the number of TFs with distinct binding site motifs are much smaller, since the TFs with similar DBDs usually have similar binding site motifs (Ambrosini et al., 2020). The most obvious exception to this general rule is the TF class C2H2 zinc finger {2.3} (Lambert et al., 2018).

**Fig. 1.**
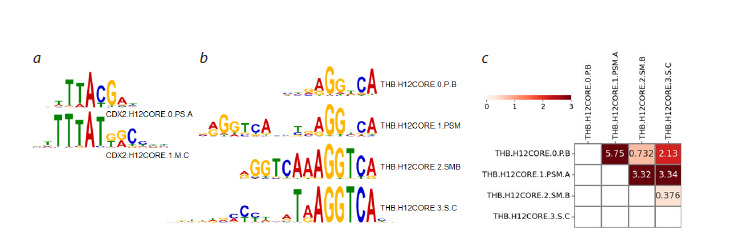
Similarity of different binding site motifs representing individual TFs. a, b – two/four binding site motifs of CDX2 / THB TFs from the Homeo domain factors {3.1} / Nuclear receptors with C4 zinc fingers {2.1} classes. For each motif, the Hocomoco DB identifier is indicated (Vorontsov et al., 2024). The PWM motif model logo represents nucleotide frequencies at positions as letter heights (Schneider, Stephens, 1990); c – motif similarity estimates calculated by the TomTom tool (Gupta et al., 2007) for four TFBS motifs of THB TF, the color reflects the significance of the similarity, −Log10[ p-value].

The presence of two or more structurally distinct binding site motifs for a single TF is widespread across various TF classes (Vorontsov et al., 2024). This is explained by the ability of certain TFs to bind only as dimers of related TFs (for example, TF pairs from the classes Basic helix-loop-helix factors (bHLH) {1.2}, or Basic leucine zipper factors (bZIP) {1.1}), or as a dimer or monomer (for example, TFs from the class Nuclear receptors with C4 zinc fingers {2.1}) (Amoutzias et al., 2008). Commonly, TFBS motifs of related TFs from the same class or family exhibit a high to moderate degree of similarity depending on the position of the class, family, or subfamily in the TFClass/Plant-TFClass hierarchy. However, even among the TFBS motifs of the same TF, a certain variety of structural variants can be observed. For example, for TF CDX2 (Homeo domain factors {3.1} class) and THB (Nuclear receptors with C4 zinc fingers {2.1} class), there are two and four motifs in Hocomoco (version 12), respectively. The two TFBS motifs of CDX2 TF are not significantly similar (p-value > 0.001, Gupta et al., 2007) (Fig. 1a), significant similarity is also absent in three of the six possible pairs of the four THB binding site motifs (Fig. 1b, c). It can be assumed that more often families or subfamilies, rather than TF classes, represent significantly similar motifs (Nagy G., Nagy L., 2020; de Martin et al., 2021; Zenker et al., 2025). We study this issue in more detail in this work.

The most important step in the analysis of ChIP-seq data, de novo motif search, reveals a list of enriched motifs for ChIP-seq peaks. For the PWM motif model, each motif is a matrix of nucleotide frequencies, and it is necessary to determine a list of known TFs from DBs, such as Jaspar (Rauluseviciute et al., 2024), Hocomoco (Vorontsov et al., 2024) or Cis-BP (Weirauch et al., 2014), having significantly similar motifs of binding sites of known TFs. However, in addition to the dependence of the number of binding site motifs on the DBD structure of a TF, TFs are extremely unevenly distributed in superclasses, classes, and even families. In the most complete human/mouse DB of TFBS motifs (Hocomoco, version 12, Vorontsov et al., 2024), the five largest TF classes represent about 75 % of all motifs (1,082 of 1,443): C2H2 zinc finger factors {2.3}, Homeo domain factors {3.1}, Basic helix-loop-helix factors (bHLH) {1.2}, Nuclear receptors with C4 zinc fingers {2.1}, and Basic leucine zipper factors (bZIP) {1.1}. The ten largest classes comprise about 90 % of all motifs (1,303 out of 1,443). The eight largest TF families from a total of four classes represent more than 51 % (742 out of 1,443) of all TFBS motifs: More than 3 adjacent zinc fingers {2.3.3}, HOX-related {3.1.1}, Multiple dispersed zinc fingers {2.3.4}, Paired-related HD {3.1.3}, NK-related {3.1.2}, Three-zinc finger Kruppel-related {2.3.1}, Talrelated {1.2.3}, and Ets-related {3.5.2}. A recent analysis of 1,725 TFs of the model plant Arabidopsis thaliana revealed about 40 % of them (686) with available TFBS motifs; the inclusion of TFBS motifs for 92 TFs from other plants showed an extremely limited vocabulary of only 74 distinct plant TFBS motifs (Zenker et al., 2025).

Very often, an enriched motif from the results of a de novo motif search has a high similarity to the TFBS motifs of known TFs from one or more families of the same class, or even an entire class falls into the list of TF candidates. The result is a list of several dozen TFs, and choosing a specific TF among them is not an easy task. Such long lists of TF candidates may complicate the identification of TFs most likely associated with enriched motifs. However, this complexity can be reduced by the systematic analysis of the similarity of the binding site motifs of TFs classified by the hierarchy levels from the TFClass DB. To date, for cognate TFs of a given structure of a DBD (class, family and subfamily), it has not been determined which of these levels is sufficient to identify a set of TFs with significantly similar binding site motifs. To solve this issue, one needs to find a set of certain arrays (or branches) of several consecutive levels of the TFClass hierarchical classification, for which the TFBS motifs are significantly similar. This approach is able to further systematize the hierarchical classification of TFs, adapt it to apply to the results of a de novo motif search. The resulting refined TF hierarchy will reflect the similarity of DBDs of TFs and the similarity of TFBS motifs.

We propose to include the annotation of the branches of similar binding site motifs of known TFs in a standard protocol of de novo motif search applied to the results of genome-wide mapping of TFBS in vivo, for example, using ChIP-seq technology. The application of branches can notably simplify the analysis of enriched TFBS motifs. The TF branches connect the generally accepted units of the hierarchical classification of TFs by DBDs, namely superclasses, classes, families, subfamilies (Wingender, 1997, 2013; Wingender et al., 2013, 2015, 2018) to the similarity of TFBS motifs (Gupta et al., 2007).

## Materials and methods

Input data and parameters. The input data are sets of TFBS motifs; each motif is represented by a nucleotide frequency matrix, an identifier and a TF name; for each TF, its superclass, class, family and subfamily (if any) are indicated, according to the TFClass DB (Wingender et al., 2013, 2015, 2018). TFBS motifs for human Homo sapiens and fruit fly Drosophila melanogaster were extracted from Hocomoco (version 12, https://hocomoco.autosome.org/) (Vorontsov et al., 2024) and Jaspar https://jaspar.elixir.no/ (Rauluseviciute et al., 2024). Both DBs construct TFBS motifs based on in vivo massive sequencing data (e. g. ChIP-seq), and in vitro ones (e. g. HT-SELEX). TFBS motifs are nucleotide frequency matrices consistent with the traditional PWM model. In both DBs, TF classification is applied according the DBD structure by hierarchy levels of superclass, class, family, subfamily and TF (TFClass DB, Wingender, 2013; Wingender et al., 2013, 2015, 2018). We selected for analysis the classes amounting to at least 50 TFBS motifs: seven / two classes for human / Drosophila TFs, see the Table.

**Table 1.**
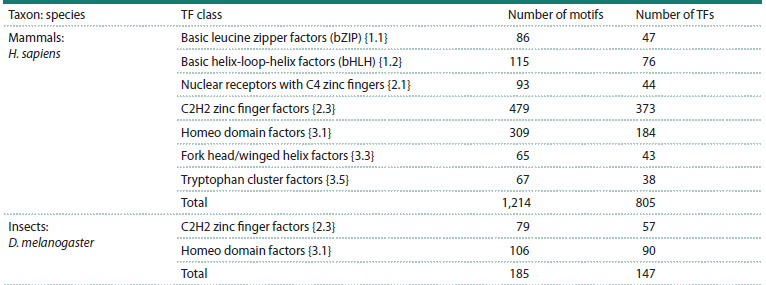
TFBS motif sets from the Hocomoco and Jaspar DBs used in analysis

Similarity metric of two TFs. We applied the TomTom tool (Gupta et al., 2007) to assess the significance of similarity (p-value) in pairs of TFBS motifs, the parameter of the motif comparison function was the Pearson correlation coefficient. Two TFBS motifs were considered similar if the significance level reached the threshold, −Log10[ p-value] > Thr = 3.

We define the similarity metric for a pair of TFs based on their binding site motifs according to the distribution of similarity in all possible pairs of binding site motifs of one and another TF, since TFs can have one or more binding site motifs. Let two TFs X/Y have NX/ NY motifs, {Mi}, 1 ≤ i ≤ NX and {Mj}, 1 ≤ j ≤ NY, correspondingly. The distribution of similarity estimates in a pair of these TFs based on their binding site motifs includes NX × NY pairs of motifs. Let the similarity Score(Mi, Mj) of motifs Mi and Mj be given by TomTom (Gupta et al., 2007) as the logarithm of the significance p-value:

**Formula. 1.**
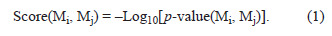
Formula1

Then for two TFs X and Y, the similarity metrics ScoreX,Y will be defined as follows:

**Formula. 2.**
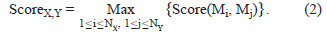
Formula2

If this metrics ScoreX,Y (2) exceeds the pre-defined threshold Thr, then TFs X and Y can be considered significantly similar in their binding site motifs. For one TF, the heterogeneity of binding site motifs is estimated as the median (the second quartile, Q2) of the distribution over all possible pairs of binding site motifs of that TF:

**Formula. 3.**
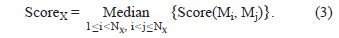
Formula3

Similarity metric of two sets of TFs. Let a class have a family A with NA TFs. The distribution of all possible TF pairs in this family includes NA × (NA – 1)/2 variants. Let a family B from the same class have NB TFs. The distribution of all possible TF pairs of families A and B includes NA × NB variants. For both the intra-family and inter-family cases, for all TF pairs, the similarity estimates are calculated by the formula (2). Likewise, pairs of subfamilies in the same family and pairs of classes in the same superclass are considered.

For the obtained distribution of similarity estimates, it is possible to calculate five similarity metrics for two sets of TFs: minimum (Min), quartiles Q1, Q2 (median) and Q3, and maximum (Max). Min/Max metrics indicate the choice of the minimum/maximum values, and quartile metrics indicate the value of the corresponding fraction of the entire distribution. For example, the Q2 (median) metric for two sets of TFs reflects a level of similarity of 50 % of all possible TF pairs from these sets. Let the first {X} and second {Y} sets have K and T TFs, 1 ≤ k ≤ K, 1 ≤ t ≤ T, then based on the distributions of the similarity values in TF pairs calculated by the formula (2) {ScoreX(k),Y(t)}, the similarity metric Score{X},{Y} of the two TF sets is calculated as follows:

**Formula. 4.**
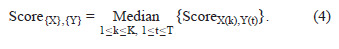
Formula4

Definition of the branch in the TF hierarchical classification. If the similarity score of two sets of TFs based on their binding site motifs exceeds the predetermined threshold Thr, then these TFs can be referred to the same branch. Next, consider the median metric (4). For example, an entire class can belong to the same branch if more than half of all its possible TF pairs are similar in terms of binding site motifs. Although it is possible that certain families of a class do not show significant similarity, with a probability of more than 50 %, an arbitrary pair of TFs from this class shows the significant similarity of binding site motifs.

To perform cluster analysis and construct trees reflec- ting the similarity of TFs based on the TFBS of the sister classes of the same superclass, the sister families of the same class, etc., we used the UPGMA algorithm scheme (unweighted pair group method with arithmetic mean) (Sokal, Michener, 1958). During the classification, we applied the median metric (Q2, formula (4)) described above to evaluate any pair of objects.

To search for branches, the analysis starts at the superclass level, and continues at lower levels of the hierarchy: the class, family, subfamily, or TF. First, the TF similarity metric is calculated within a given hierarchy level, for example, a class, as well as for all families of this class. This gives a list of families with similarities exceeding the threshold Thr. All such families initially refer to different branches; to analyze the remaining families, we need to go to a lower level. Then the TF similarity metrics are calculated for all possible pairs of the sister families of this class. This gives the similarity matrix for families of the class. The diagonal values of the matrix show the similarities within each family and those above the diagonal provide the similarities for all pairs of different families. Next, we select a pair of families with the highest similarity. If this similarity exceeds the threshold, then a pair of such families (branches) are joined into one branch. After that, the similarities in all pairs of updated branches are recalculated. Calculations continue as long as there are pairs of branches that allow joining based on their similarity. In such a way we can gradually descend to the lower levels and reach the level of TF.

The similarity of the binding site motifs of single TFs is analyzed separately (see formula (3)), although, obviously, this analysis takes place inside one branch, since according to formulas (2) and (4), each branch for any TF contains all its binding site motifs, and we can only note TFs (Fig. 1) having significantly different binding site motifs.

The purpose of the whole analysis is to sequentially find such sets of TFs (for example, for a class, this is a list of family clusters), for which the metric (4) exceeds the given threshold Thr, and the list for each of the branches includes as many elementary classification units as possible.

TF superclasses are heterogeneous enough in the similarity of binding site motifs since each superclass splits into multiple branches. A branch in the TFClass hierarchy is defined as the maximum possible set of TFs from the highest class level to the lowest level (in practice, this is a class, family, subfamily, TF), such that in this set for the majority of TF pairs there is a significant similarity of TFs based on their binding site motifs, according to the similarity metric (4).

A branch may include one or more sister classification units: • a whole class, • one or more families of the same class, • one or more subfamilies of the same family, • one or more TFs of the same subfamily.

**Fig. 2.**
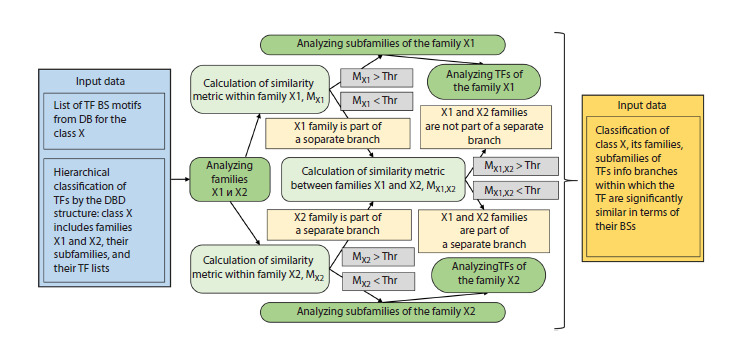
Scheme of analysis to determine branches of similar motifs of TFBS. The scheme shows in detail the stage of analysis of one class X consisting of two families X1 and X2. The blue color shows the input data, dark green – analysis stages, light green – similarity metric calculations, gray – verification of similarity conditions for motifs, light yellow – intermediate results, dark yellow – final results. The scheme discloses the analysis of two families X1 and X2 of class X. The analysis of subfamilies of these families and the analysis of TFs in each of the subfamilies are performed similarly to the analysis of families X1 and X2, as described in the text.

The final result of the analysis is the determination of the set of all branches, within each of the branches, the metric (4) indicates significant similarity of TFs based on TFBS motifs. Figure 2 is a scheme of the analysis used in the work.

## Results

**Fig. 3.**
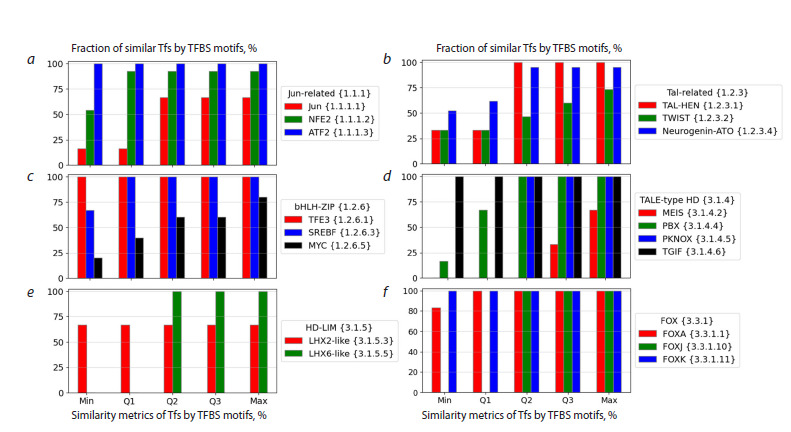
Fraction of significantly similar TFs based on the binding site motifs for subfamilies of different families using the five similarity metrics: Min, Q1, Q2, Q3, and Max. a–e, and f – Jun-related {1.1.1}, Tal-related {1.2.3}, bHLH-ZIP {1.2.6}, TALE-type HD {3.1.4}, HD-LIM {3.1.5}, and FOX {3.3.1} families, respectively. Color marks subfamilies. The X axis lists TF similarity metrics; the Y axis shows the fraction of significantly similar TFs based on the binding site motifs in the subfamily. Significant similarity requires the criterion −Log10[p-value] > 3 (Tomtom tool, Gupta et al., 2007).

**Similarity of TFs in sister subfamilies of the same families** In order to start a massive analysis of different degrees of similarity of binding site motifs to cognate TFs according to the TFClass hierarchical classification, we test the TFBS motif similarity for subfamilies of individual families belonging to various TF classes. Figure 3 shows the fraction of similar TFs based on the binding site motifs within subfamilies of different families, using the five metrics Min, Q1, Q2, Q3, and Max. The Q2 metric (median) is calculated according to the formula (4), others metrics are computed likewise. By construction, among these metrics from Min to Max, the fraction of the similar TFBS motifs is growing. However, regardless of the metric choice, some subfamilies show a lower similarity or even a complete lack of similar TFBS motifs, compared to other subfamilies. For example, for the three subfamilies of the Fox {3.3.1} family, the values of the Q2 metric are close to 100 % (Fig. 3f ), and for the subfamilies TWIST {1.2.3.2}/MEIS {3.1.4.2} of the families Tal-related {1.2.3}/TALE-type HD {3.1.4}, respectively, these values are less than 50 % (Fig. 3b, d ).

Thus, the similarity of TFs based on binding site motifs can vary significantly across the subfamilies of the same families. Obviously, the same conclusion can be drawn for the families of the same classes. Further, in the analysis, the median metric (Q2) (4) was used to assess the similarity of the two sets of TFs, since the meaning of its application is the most transparent compared to the Min, Q1, Q3, and Max metrics. Hereinafter, the value of the Q2 metric is called “similarity”.

**Similarity analysis of human TFs**

**Fig. 4.**
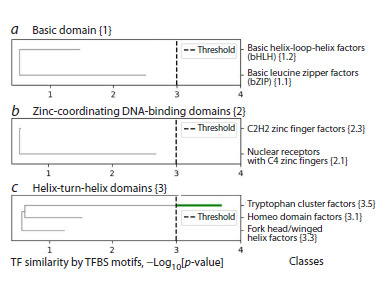
Similarity of TFs based on binding site motifs in the largest classes of the three largest human superclasses a, b, and c – class TF trees for the superclasses Basic domain {1}, Zinccoordinating DNA-binding domains {2}, and Helix-turn-helix domains {3}. The X axis reflects the value of the Q2 metric, the dash line shows its threshold value 3. The green color shows the class Tryptophan cluster factors {3.5}, which forms a separate branch, and the gray color indicates paths, the Q2 metric values of which are less than the threshold. Horizontal line break marks the value of the Q2 metric.

Figure 4 shows the human TF similarity trees based on binding site motifs for the main classes of the three largest superclasses: Basic domain {1}, Zinc-coordinating DNAbinding domains {2} and Helix-turn-helix domains {3}. Of all the classes, only one class Tryptophan cluster factors {3.5} shows the significant similarity of TFs based on their binding sites motifs (similarity 3.68). The classes Basic leucine zipper factors (bZIP) {1.1} and Nuclear receptors with C4 zinc fingers {2.1} reach the similarity values of 2.51 and 2.68, respectively, indicating a trend towards significant similarity. The classes Fork head/winged helix factors {3.3}, Homeo domain factors {3.1} and Basic helix-loop-helix factors (bHLH) {1.2} show lower similarity values of 1.14, 1.42 and 1.47. The lowest similarity of TFs based on the binding site motifs is found for the class C2H2 class zinc finger factors {2.3} (0.44); this class is the largest in human, allowing the greatest variability in the structure of TFs (Najafabadi et al., 2015; Lambert et al., 2018, 2019).

Therefore, to identify branches within all classes except the class Tryptophan cluster factors {3.5}, it is necessary to proceed to the analysis of their families. Next, we will separately consider each of the three superclasses in more detail.

**Fig. 5.**
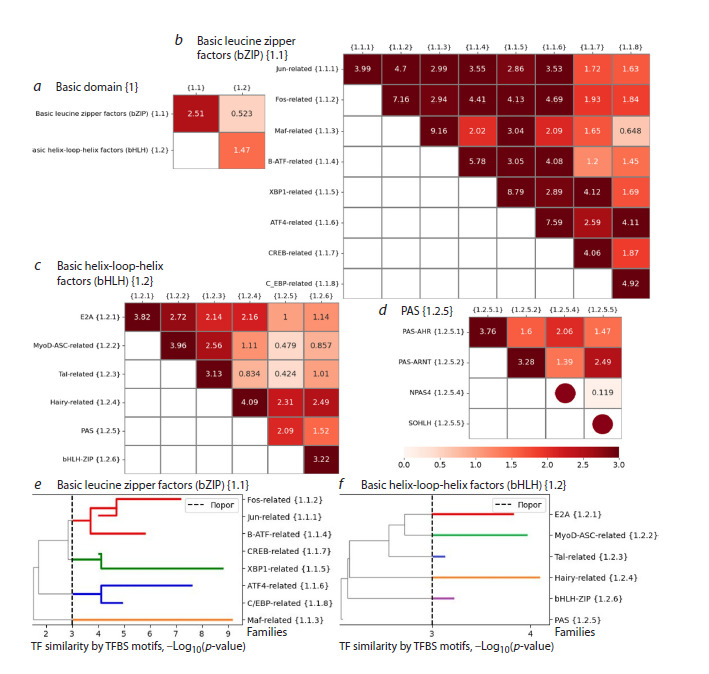
TF similarity based on binding site motifs for the Basic domain {1} superclass a–d – heatmaps for classes of the superclass, for families of the Basic leucine zipper factors (bZIP) {1.1}/Basic helix-loop-helix factors (bHLH) {1.2} classes and for subfamilies of the PAS {1.2.5} family of the Basic helix-loop-helix factors (bHLH) {1.2} class. A brown circle on the heatmap diagonal means that the subfamily has only one TF with one TFBS motif. The color reflects the value of the Q2 similarity metric. Here and further to the right of each heatmap are the names of classes/families/subfamilies along with their numerals, and above are only numerals; e and f – family trees for the classes Basic leucine zipper factors (bZIP) {1.1} and Basic helix-loop-helix factors (bHLH) {1.2}. The Y axis reflects the value of the Q2 metric, the dash line shows its threshold value 3. All colors except gray reflect individual branches, and gray highlights paths, the Q2 metric value of which is less than the threshold. A horizontal line break marks the value of the Q2 metric for the family. The Jun-related {1.1.1} family (e) has a lower similarity of 3.99 (b) than the similarity of the union of Jun-related {1.1.1} and Fos-related {1.1.2} families, so the direction of the path of the Jun-related {1.1.1} family from the junction point of these two families changes to the opposite

The first superclass has two large classes, Basic leucine zipper factors (bZIP) {1.1} and Basic helix-loop-helix factors (bHLH) {1.2}; the similarity of TFs based on binding site motifs between these classes is very low (0.523, Fig. 5a). The similarity of TFs within each class is noticeably higher, but the Basic leucine zipper factors (bZIP) {1.1} class has distinctly more similar TFs (2.51) than the Basic helix-loop-helix factors (bHLH) {1.2} class (1.47).

There are eight families in the Basic leucine zipper factors (bZIP) {1.1} class (Fig. 5b, e): from Jun-related {1.1.1} to C/EBP-related {1.1.8}. Each family of the class has one or more other families with significantly similar TFs based on binding site motifs. As a result, all families fall into four branches (Fig. 5e); there are two branches of two families (XBP1-related {1.1.5} and CREB-related {1.1.7}, ATF4- related {1.1.6} and C/EBP-related {1.1.8}), and the branches of one (Maf-related {1.1.3}) and three families (Jun-related {1.1.1}, Fos-related {1.1.2}, B-ATF-related {1.1.4}).

In the Basic helix-loop-helix factors (bHLH) {1.2} class, within each of the families, with the exception of one (PAS {1.2.5}), TFs have significant similarities based on the binding site motifs (Fig. 5b, values on the diagonal), but there are no significant similarities between TF families based on the binding site motifs. Therefore, each of the families, with the exception of the PAS {1.2.5} family, forms a separate branch (Fig. 5f). The PAS family {1.2.5} is divided into four branches {1.2.5.1}, {1.2.5.2}, {1.2.5.3} and {1.2.5.4} by four subfamilies (Fig. 5d).

**Fig. 6.**
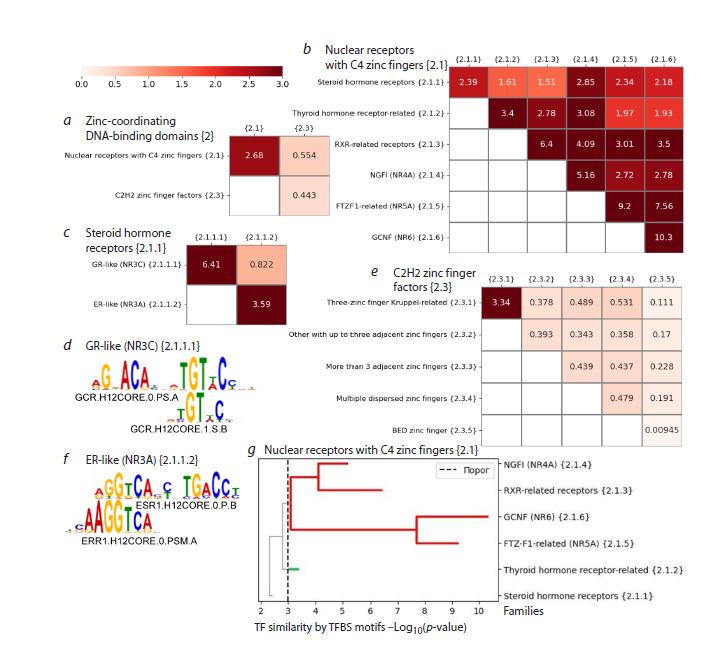
Similarity of TFs based on binding site motifs for the superclass Zinc-coordinating DNA-binding domains {2}. a, b, c and f – heatmaps for classes of the superclass, for families of the class Nuclear receptors with C4 zinc fingers {2.1}, for subfamilies of the family Steroid hormone receptors {2.1.1} of the class Nuclear receptors with C4 zinc fingers {2.1} and for families of the class C2H2 zinc finger factors {2.3}; d, e – examples of TF binding site motifs from the GR-like (NR3C) {2.1.1.1}/ER-like (NR3A) {2.1.1.2} subfamilies of the family Steroid hormone receptors {2.1.1}; g – family tree for the Nuclear receptors with C4 zinc fingers {2.1} class. The Y axis implies the value of the Q2 metric, the dash line means the threshold value 3. Red and green colors reflect separate branches, and paths are highlighted in gray, if the respective value of the Q2 metric is less than the branch threshold. Horizontal line break marks the value of the Q2 metric.

The second superclass has two large classes Nuclear receptors with C4 zinc fingers {2.1} and C2H2 zinc finger factors {2.3}, the similarity of TFs based on binding site motifs between these classes is very low (0.554, Fig. 6a). In the Nuclear receptors with C4 zinc fingers {2.1} class, TFs have the similarity only slightly below the threshold (2.68), and the TF similarity in the class C2H2 zinc finger factors {2.3} is very low (0.443).

In the class Nuclear receptors with C4 zinc fingers {2.1} (Fig. 6b), only one family, Steroid hormone receptors {2.1.1}, has a similarity of TFs 2.39 below the threshold. This family is divided into two branches according to the two subfamilies: GR-like (NR3C) {2.1.1.1} and ER-like (NR3A) {2.1.1.2} (Fig. 6c). The similarity of TFs between these subfamilies is low (0.822), and within each subfamily, it is high (6.41 and 3.59). TFBS motifs from these related subfamilies have a similar structure: TFs of both subfamilies can bind DNA as monomers or as dimers formed by an inverted repeat (Nagy G., Nagy L., 2020), but regardless of this, the monomeric subunits in TFBS motifs of the GR-like (NR3C) {2.1.1.1} (Fig. 6d) and ER-like (NR3A) {2.1.1.2} subfamilies (Fig. 6e) are clearly distinct. The Thyroid hormone receptor-related {2.1.2} family forms a separate branch, since the similarity of its TFs with the TFs of four of the five other families is below the threshold 3 (Fig. 6b, g). Four families from the RXR-related receptors {2.1.3} to GCNF (NR6) {2.1.6} form one branch: Figure 6f shows the tree dividing the Nuclear receptors with the C4 zinc fingers {2.1} class into branches by families.

In the C2H2 zinc finger factors {2.3} class (Fig. 6f ), only one family, Three-zinc finger Kruppel-related {2.3.1}, forms a separate branch. To determine the branches of the other four families of the class, we need to go down to the levels of subfamilies or TFs, see the list of all branches of the C2H2 zinc finger factors {2.3} class in Table S11.


Supplementary Materials are available in the online version of the paper: https://vavilov.elpub.ru/jour/manager/files/Suppl_Levitsky_Engl_29_7.pdf


**Fig. 7.**
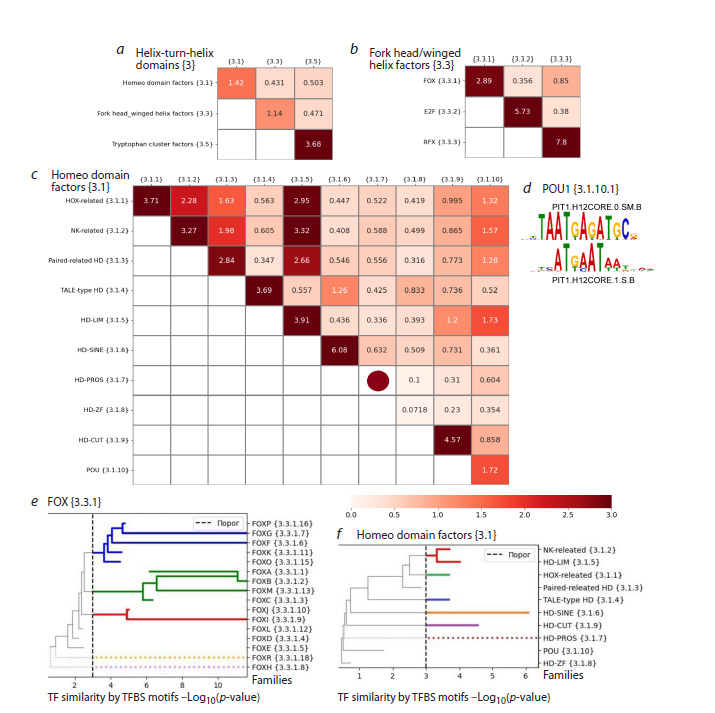
Similarity of TFs based on binding site motifs for the superclass Helix-turn-helix domains {3}. a–c – heatmaps for classes of the superclass, for families of the classes Fork head/winged helix factors {3.3} and Homeo domain factors {3.1}. The brown circle on the heatmap diagonal means that the family has only one TF with one binding site motif. The color reflects the value of the Q2 similarity metric; d – logo of two binding site motifs of TF PIT1 from the subfamily POU1 {3.1.10.1}; e and f – trees for subfamilies of the FOX {3.3.1} family and for families of the Homeo domain factors {3.1} class. The Y axis reflects the value of the Q2 metric, the dash line shows its threshold value 3. Dotted lines mean a single TF with one binding site motif in the current family or subfamily. All colors except gray reflect individual branches, and gray indicates paths, the Q2 metric value of which is less than the branch threshold. Horizontal line break marks the value of the Q2 metric. The subfamily FOXA {3.3.1.1} (e) has a lower similarity of 6.22 (Fig. S1) than the similarity of the union of the subfamilies FOXA {3.3.1.1} and FOXB {3.3.1.2}, so the direction of the path of the subfamily FOXA {3.3.1.1} from the junction point of these two subfamilies changes to the opposite.

The third superclass includes three large classes Homeo domain factors {3.1}, Fork head/winged helix factors {3.3}, and Tryptophan cluster factors {3.5}. The similarity between TFs of different classes based on binding site motifs is very low in all three possible pairs of classes (Fig. 7a, cells above the diagonal). Similarity of TFs within each of the classes Homeo domain factors {3.1}, Fork head/winged helix factors {3.3} is medium, 1.42 and 1.12. The class Tryptophan cluster factors {3.5} forms one branch (Fig. 4).

In the class Fork head/winged helix factors {3.3}, two families E2F {3.3.2} and RFX {3.3.3} represent two separate branches, and the similarity of TFs of the FOX family {3.3.1} almost reaches the threshold (similarity value 2.89, Fig. 7b). A vivid illustration of the correctness of the division of the Fork head/winged helix factors {3.3} class into three families (Fig. 7b) is a noticeable excess of the similarity of TFs within families (three values on the diagonal) in relation to the similarity of TFs between families (three values above the diagonal).

Among the 16 subfamilies of the FOX family {3.3.1} (Fig. 7e), only three subfamilies FOXD {3.3.1.4}, FOXH {3.3.1.5} and FOXL {3.3.1.12} achieved TF similarity below the threshold 3: 2.19, 2.48 and 2.17, respectively. Four, five and two subfamilies form separate branches (Fig. 7e). There are two subfamilies, FOXH {3.3.1.8} and FOXR {3.3.1.18}, with low similarity of TFs based on binding site motifs with other subfamilies and between themselves (Fig. S1).

Two families (NK-related {3.1.2} and HD-LIM {3.1.5}) of the Homeo domain factors {3.1} class merge into one branch; each of five HOX-related {3.1.1}, TALE-type HD {3.1.4}, HD-SINE {3.1.6}, HD-PROS {3.1.7} and HD-CUT {3.1.9} families represents a separate branch (Fig. 7c, f ). To find branches for the remaining families Paired-related HD {3.1.3}, HD-ZF {3.1.8} and POU {3.1.10}, it is necessary to proceed to the subfamily level (Fig. S2, Table S1). The Paired-related HD {3.1.3} family is divided into two separate branches, combining 12 and 6 subfamilies (Fig. S2a, Table S1). The HDZF {3.1.8} family is divided into two branches according to two subfamilies, ZEB {3.1.8.3} and ZHX {3.1.8.5} (Fig. S2b). Three subfamilies POU2 {3.1.10.2}, POU3 {3.1.10.3} and POU5 {3.1.10.5} merge into one branch. The subfamily POU1 {3.1.10.1} is represented by one TF PIT1 with two significantly dissimilar TFBS motifs PIT1.H12CORE.0.SM.B and PIT1.H12CORE.1.S.B (Fig. 7d). The remaining three subfamilies POU4 {3.1.10.4}, POU6 {3.1.10.6} and HNF1- like {3.1.10.7} of the family POU {3.1.10} form separate branches (Fig. S2c).

The full list of branches for the seven largest TF classes Basic leucine zipper factors (bZIP) {1.1}, Basic helix-loophelix factors (bHLH) {1.2}, Nuclear receptors with C4 zinc fingers {2.1}, Homeo domain factors {3.1}, Fork head / winged helix factors {3.3} and Tryptophan cluster factors {3.5} is given in Table S1.

In general, based on the results presented in Figures 5–7 and in Figures S1, S2 and Table S1, we can conclude that often TFs of the same family already have dissimilar binding site motifs. However, this general trend is broken for some classes and families. It is most clearly violated for the largest class of human TFs C2H2 zinc finger factors {2.3} (Fig. 6f ), for which it is necessary to descend to the level of subfamilies or even to the level of TFs to determine branches.

**Similarity analysis of Drosophila TFs**

To determine how the discovered patterns of similarity in different classes of TFs depend on the choice of taxon, we conducted an analysis analogous to that carried out above for the insect taxon sufficiently distant from the mammalian taxon. According to the Jaspar DB, there are only two classes of insect TFs with more than 50 binding site motifs (see the Table). All these TFs belong to the species D. melanogaster. The results obtained for insect TFs from these two classes, C2H2 zinc finger factors {2.3} and Homeo domain factors {3.1}, are in good agreement with the results obtained above for human TFs from seven classes (Fig. 4–7).

**Fig. 8.**
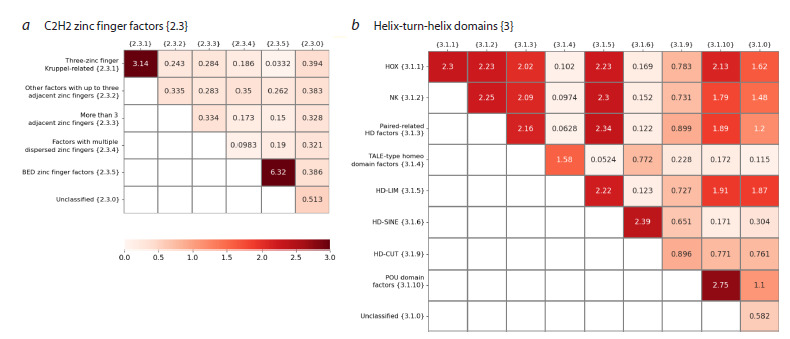
Similarity of Drosophila TFs from the two large classes based on binding site motifs. a and b – heatmaps for families of the classes C2H2 zinc finger factors {2.3} and Homeo domain factors {3.1}. The color reflects the value of the Q2 similarity metric.

In the Drosophila C2H2 zinc finger factors {2.3} class (Fig. 8a), as well as in the same class in human (Fig. 6f ), only one family, Three-zinc finger Kruppel-related {2.3.1}, has significantly similar TFs based on binding site motifs. Only TFs of one other family, BED zinc finger {2.3.5}, have very different similarity of binding site motifs (human 0.001, Drosophila 6.32). However, this family is very small: in Drosophila, it contains two almost indistinguishable binding site motifs of one TF Dref; and in human, two TFs ZBED1 and ZBED5 have clearly dissimilar to each other motifs of binding sites. The other three common families in both taxa, Other factors with up to three adjacent zinc fingers {2.3.2}, More than 3 adjacent zinc fingers {2.3.3}, Multiple dispersed zinc fingers {2.3.4}, as well as all remaining Drosophila TFs with unspecified families, assigned to the family Unclassified {2.3.0}, show extremely low similarity of TFs based on binding site motifs. In general, for both human and Drosophila TFs, the class C2H2 zinc finger factors {2.3} has TFs with very low similarity of binding site motifs (Fig. 6f, 8a).

Drosophila TFs from the Homeo domain factors {3.1} class (Fig. 8b) show slightly less similarity in terms of binding site motifs than TFs from the same human class (Fig. 7c). However, in each of these two taxa, among the eight common families, families with greater and lesser similarity of TFs based on binding site motifs are distinguished. Namely, in both taxa, TFs from four families – HOX-related {3.1.1}, NK-related {3.1.2}, Paired-related HD {3.1.3} and HD-LIM {3.1.5} – have the greatest similarity, both within and between families (Fig. 7c, f ); however, the similarity itself exceeds the value 2 for Drosophila TFs, but does not reach the threshold 3 (Fig. 8b). The remaining families have TFs that are not similar both to each other and to TFs of the above families of the class. In general, much smaller similarity in the binding site motifs of Drosophila TFs of the Homeo domain factors class {3.1} (Fig. 8b) compared with the human TFs of the same class (Fig. 8c) can be explained by the noticeably smaller number of available massive sequencing data for Drosophila TFBSs (see the Table). Another explanation is the difference in the methods for obtaining TFBS motifs in the Hocomoco and Jaspar DBs.

## Discussion

We propose a new systematic approach to refine the hierarchical classification of TFs according to the structure of DBDs by a set of branches combining TFs with similar motifs of binding sites. The similarity of the binding site motifs of known TFs can now be evaluated with various experimental massive sequencing technologies, including in vitro HT-SELEX and in vivo ChIP-seq data, for example, experimental results for different tissue conditions and developmental stages.

Estimates of the total numbers of human/Drosophila TFs are 1,659/651 (AnimalTFDB, Shen et al., 2023). The Hocomoco DB (version 12) for human and the Jaspar DB for Drosophila annotated 1,443 TFBS motifs for 949 TFs and 334 TFBS motifs for 273 TFs. Hence, although the ratios of the number of TFs with known binding site motifs to the estimates of the total numbers of TFs for human and Drosophila are close (57 and 51 %), on average, one TF accounts for 1.52/1.22 annotated binding site motifs for human (Hocomoco)/Drosophila (Jaspar). In accordance with this, the GTRD (Kolmykov et al., 2021) provides data on 21988/3027 ChIP-seq experiments for 1,531/595 human/Drosophila TFs. Therefore, the diversity of structural types of TFBS motifs has already been studied markedly better in human than in Drosophila.

The possible correspondence of the enriched motifs from the results of a de novo motif search to binding sites of target or partner TFs complicates the task of analyzing TF binding data in vivo. In vitro massive sequencing data, such as HT-SELEX or DAP-seq, reflect only the direct binding of target TFs, and completely exclude the cooperative binding of target TFs to any partner TFs and indirect binding of target TFs. Therefore, the nucleotide binding specificity of target TFs in vitro can determine only a fraction of their binding loci in vivo. In vivo TFBS sequencing data reflect the main cooperative mechanism of target TF binding to genomic DNA, including its interactions with various partner TFs (Morgunova, Taipale, 2017). This complicates the connection of enriched de novo motifs to specific partner TFs.

The variability of TFBS motifs derived from the systematization of their modern massive sequencing data reflects the diversity of the structure of TF DBDs. DBDs of TFs are important for the function of the direct binding of target and partner TFs. For example, only TFs from certain classes have the ability to function as dimers of closely related TFs (Amoutzias et al., 2008). Among the ones studied here (see the Table), those are TF classes Basic leucine zipper factors (bZIP) {1.1}, Basic helix-loop-helix factors (bHLH) {1.2} and Nuclear receptors with C4 zinc fingers {2.1}. The main function of a TF, its ability to interact with genomic DNA, depends on the place of this TF in the general hierarchy of the structure of the DBDs of all TFs, that is, on a superclass, class, family and subfamily of this TF. Previously, these levels of hierarchical classification of TFs were defined by the structure of their DBDs and the alignments of amino acid sequences of DBDs of TFs (TFClass DB, Wingender, 1997, 2013; Wingender et al., 2013, 2015, 2018); notably, the similarity of TFBS motifs was not taken into account to define the hierarchy. A systematic analysis of the similarity of TFBS motifs can make the classification of TFs more efficient for the practical application at the stage of interpreting enriched motifs, the results of a de novo motif search based on massive mapping of TFBS in vivo, such as ChIP-seq.

Deducing the general topology of the branches of significantly similar TFBS motifs consists in selecting for each TF such a level of hierarchy among options of one class, one or more sister families (or subfamilies), or individual TF, so that for the TFs of the entire branch, most TF pairs have significantly similar binding site motifs. To determine the list of branches, we need the following: the hierarchical classification of TFs according to the structure of their DBDs from the TFClass/Plant-TFclass DBs; TFBS motif sets from DBs; the formula for calculating similarity in a pair of TF sets based on their binding site motifs (4). Identifying all branches along the TFClass/Plant-TFclass hierarchy will help avoid excessive detail in the output data of a de novo motif search. These misleading data and excessive information arise since for any of the individual classification units, such as a specific class, or family/subfamily, there is the variability of the TFBS motifs similarity not restricted. Initially, there were no such restrictions for DBD TFs, too (Wingender, 1997, 2013; Wingender et al., 2013, 2015, 2018).

We include TF classes with more than 50 TFBS motifs in the analysis (see the Table). Of the seven largest human classes (Fig. 4), only one, the Tryptophan cluster factors {3.5} class, shows significant similarity of TFBS motifs. For the classes Basic leucine zipper factors (bZIP) {1.1} and Nuclear receptors with C4 zinc fingers {2.1}, similarity is below the significance threshold (value 3), but is still noticeable (values between 2 and 3). Even the classes Basic helix-loop-helix factors classes (bHLH) {1.2}, Homeo domain factors {3.1} and Fork head/winged helix factors {3.3} have lower similarity (values ranging from 1 to 2). However, for the C2H2 zinc finger factors {2.3} class, the similarity value is less than 1. This low value reflects the presence of a majority of TF pairs with completely different binding site motifs in this class; approximately the same similarity values are observed between binding site motifs in any pair of TFs from different classes of the same superclass (see values in cells above the diagonal in Fig. 5a, 6a, 7a). Similar discrepancies are observed at a lower level of TF families.

For each of the classes Basic leucine zipper factors (bZIP) {1.1} and Nuclear receptors with C4 zinc fingers {2.1}, in most cases, several sister families are joined into one branch (Fig. 5e, 6g). For the classes Basic helix-loop-helix factors classes (bHLH) {1.2}, Homeo domain factors {3.1} and Fork head/winged helix factors {3.3} (Fig. 5f, 7b, f ), partitioning into branches is closer to the level of families. The level of families is clearly not enough to distinguish branches in the C2H2 zinc finger factors {2.3} class (Fig. 6f ). So, our analysis confirms clear differences in the variability of binding site motifs for the largest classes of human TFs (Fig. 4–7) (Lambert et al., 2018; Ambrosini et al., 2020). A concordant trend is also observed for the motifs of binding sites from the two largest classes of insect TFs (Fig. 8). This conclusion is in good agreement with the results of a massive comparison of the nucleotide specificity of orthologous human and Drosophila TFs, where it was found that, in general, human and Drosophila TFBS motifs showed a high level of conservation (Nitta et al., 2015). Later, a detailed analysis refined this finding. The analysis of similarity of binding site motifs of TFs from various classes in different eukaryotic taxa in lines of multicellular animals and higher plants showed that conservation in both animal and plant lineages is highly dependent on the TF class (Lambert et al., 2019). For example, almost half of the dissimilar binding site motifs of orthologous human and Drosophila TFs belonged to the C2H2 zinc finger factors {2.3} class, which is consistent with the results of our analysis (Fig. 6f, 8a). The analysis (Lambert et al., 2019) also showed that for some orthologous TFs of Drosophila and human, the similarity extended even to the level of subtle dinucleotide frequency preferences in the TFBS motifs.

We have also concluded that among the large classes of TFs, the class C2H2 zinc finger factors {2.3} has TFs with the most variable binding site motifs in human and Drosophila (Fig. 6f, 8a). Compared to the class C2H2 zinc finger factors {2.3}, both taxa have less variable TFBS motifs in the class Homeo domain factors {3.1}. However, for TFs of the class Homeo domain factors {3.1}, a greater variability of binding site motifs is found in Drosophila compared to human (Fig. 7c, 8b). This result may reflect differences in the TFBS motifs processing pipelines in the Hocomoco and Jaspar DBs.

In the Hocomoco DB, binding site motifs for each individual TF reflect data from several massive sequencing experiments for this TF (Kolmykov et al., 2021; Vorontsov et al., 2024), such as ChIP-seq and HT-SELEX; for example, often even available data of human and mouse species are combined. The goal of the analysis in the Hocomoco DB is to integrate all available data on the binding sites of individual TFs. This allows identifying as much as possible different structural types of motifs of the binding sites of each TF. The Jaspar DB has a simpler way of presenting each of the motifs with a separate experiment, which can be considered justified since there is still only a small amount of data on individual TFs. For insect TFBS motifs, an analysis similar to that carried out to obtain Hocomoco DB TFBS motifs has not yet been carried out, which is partly due to the significantly smaller pool of massive sequencing data available (Kolmykov et al., 2021; Rauluseviciute et al., 2024). It can be assumed that the approach of the Hocomoco DB compared to that of the Jaspar DB most likely reflects a greater number of minor motifs of binding sites for each of the TFs, which may contribute to a greater similarity of motifs deduced in our study, according to the formulas (2) and (4). Nevertheless, regular updates and an increase in the amount of data on known TFBS motifs in both Hocomoco and Jaspar DBs in recent years (Vorontsov et al., 2024; Rauluseviciute et al., 2024) indicate that the classification of TFBS motifs may be refined in the near future.

In general, based on our results, we can conclude that for both taxa, mammals and insects, marked differences in the similarity of binding site motifs of TFs from large classes and their families make it difficult to use the standard TFClass DB terminology, which includes TF classes, families and subfamilies, to describe the variability of TFBS motifs. Therefore, a more efficient detection of functionally involved TFs by massive sequencing of TFBS in vivo requires a systematic analysis of the similarity of binding site motifs of known TFs in order to define the variability of TFBS motifs within different elementary classification units from classes to individual TFs.

In the future, a more extensive analysis of the similarity of binding site motifs within all classes, families, subfamilies of TFs and individual TFs in model species of mammals, insects and higher plants can be a solid basis for more efficient definition of TFBS motifs from ChIP-seq massive sequencing data. Based on the performed massive analysis, we suggest that the results of a de novo motif search, for the detected enriched motifs, should indicate not only the names of TFs with the names of the class/family/subfamily attached to them, but also the branches of the hierarchical classification of TFs defined in our study. These branches are composite classification units that integrate several consecutive hierarchy levels. Each branch represents, within the framework of united multi-level classification of TFs by similarity and DBD alignment, a set of TFs with significantly similar binding site motifs.

## Conclusion

In this work, we present the approach for a systematic analysis of the similarity of the motifs of binding sites of known TFs based on a multi-level hierarchy of TFs according to the structure of DBDs from the TFClass DB, which includes the levels of superclasses, classes, families, subfamilies and individual TFs. In the general hierarchy, we determined for the large classes of mammalian (human) and insect (fruit fly) TFs the common trees of branches with TFs significantly similar in motifs of binding sites. Our analysis included seven mammalian TF classes, Basic leucine zipper factors (bZIP) {1.1}, Basic helix-loop-helix factors (bHLH) {1.2}, Nuclear receptors with C4 zinc fingers {2.1}, C2H2 zinc finger factors {2.3}, Homeo domain factors {3.1}, Fork head/winged helix factors {3.3} and Tryptophan cluster factors {3.5}, and two classes of insect TFs, C2H2 zinc finger factors {2.3} and Homeo domain factors {3.1}. We have shown that both for the taxon of mammals and for the taxon of insects, the similarity of the binding site motifs is noticeably different among TFs from distinct classes. A systematic analysis of the similarity of the binding site motifs of structurally related TFs, determined according to the hierarchical classification, allowed to determine the levels of the hierarchy (classes, families, subfamilies, TFs), starting from which and lower in the hierarchy the binding site motifs of known TFs become significantly similar. In addition to improving the identification of involved TFs from the results of a de novo motif search, leading to more efficient identification of gene regulation mechanisms, our results may refine the hierarchical classification of TFs by their DBDs. We do not redefine the classification of TFs by elementary units from the class, family and lower in the hierarchy; we provide additional information about the similarity of the TFBS motifs, which reflects the main function of TFs, the function of specific binding to the DNA sequence, which, of course, should more accurately distinguish different TFs.

## Conflict of interest

The authors declare no conflict of interest.
